# A Genome-Wide Association Study in Chronic Obstructive Pulmonary Disease (COPD): Identification of Two Major Susceptibility Loci

**DOI:** 10.1371/journal.pgen.1000421

**Published:** 2009-03-20

**Authors:** Sreekumar G. Pillai, Dongliang Ge, Guohua Zhu, Xiangyang Kong, Kevin V. Shianna, Anna C. Need, Sheng Feng, Craig P. Hersh, Per Bakke, Amund Gulsvik, Andreas Ruppert, Karin C. Lødrup Carlsen, Allen Roses, Wayne Anderson, ICGN Investigators, Stephen I. Rennard, David A. Lomas, Edwin K. Silverman, David B. Goldstein

**Affiliations:** 1 Genetics Division, GlaxoSmithKline Research and Development, Research Triangle Park, North Carolina, United States of America; 2 IGSP Center for Population Genomics and Pharmacogenetics, Duke University, Durham, North Carolina, United States of America; 3 Brigham and Women's Hospital, Boston, Massachusetts, United States of America; 4 University of Bergen, Bergen, Norway; 5 Genetics Research Centre, Munich, Germany; 6 Ullevaal University Hospital and the Faculty of Medicine, University of Oslo, Oslo, Norway; 7 Deane Drug Discovery Institute, Duke University, Durham, North Carolina, United States of America; 8 Department of Internal Medicine, University of Nebraska Medical Center, Omaha, Nebraska, United States of America; 9 Cambridge Institute for Medical Research, Cambridge, United Kingdom; University of Oxford, United Kingdom

## Abstract

There is considerable variability in the susceptibility of smokers to develop chronic obstructive pulmonary disease (COPD). The only known genetic risk factor is severe deficiency of α_1_-antitrypsin, which is present in 1–2% of individuals with COPD. We conducted a genome-wide association study (GWAS) in a homogenous case-control cohort from Bergen, Norway (823 COPD cases and 810 smoking controls) and evaluated the top 100 single nucleotide polymorphisms (SNPs) in the family-based International COPD Genetics Network (ICGN; 1891 Caucasian individuals from 606 pedigrees) study. The polymorphisms that showed replication were further evaluated in 389 subjects from the US National Emphysema Treatment Trial (NETT) and 472 controls from the Normative Aging Study (NAS) and then in a fourth cohort of 949 individuals from 127 extended pedigrees from the Boston Early-Onset COPD population. Logistic regression models with adjustments of covariates were used to analyze the case-control populations. Family-based association analyses were conducted for a diagnosis of COPD and lung function in the family populations. Two SNPs at the α-nicotinic acetylcholine receptor (CHRNA 3/5) locus were identified in the genome-wide association study. They showed unambiguous replication in the ICGN family-based analysis and in the NETT case-control analysis with combined *p*-values of 1.48×10^−10^, (rs8034191) and 5.74×10^−10^ (rs1051730). Furthermore, these SNPs were significantly associated with lung function in both the ICGN and Boston Early-Onset COPD populations. The C allele of the rs8034191 SNP was estimated to have a population attributable risk for COPD of 12.2%. The association of hedgehog interacting protein (HHIP) locus on chromosome 4 was also consistently replicated, but did not reach genome-wide significance levels. Genome-wide significant association of the HHIP locus with lung function was identified in the Framingham Heart study (Wilk et al., companion article in this issue of *PLoS Genetics*; doi:10.1371/journal.pgen.1000429). The CHRNA 3/5 and the HHIP loci make a significant contribution to the risk of COPD. CHRNA3/5 is the same locus that has been implicated in the risk of lung cancer.

## Author Summary

There is considerable variability in the susceptibility of smokers to develop chronic obstructive pulmonary disease (COPD), which is a heritable multi-factorial trait. Identifying the genetic determinants of COPD risk will have tremendous public health importance. This study describes the first genome-wide association study (GWAS) in COPD. We conducted a GWAS in a homogenous case-control cohort from Norway and evaluated the top 100 single nucleotide polymorphisms in the family-based International COPD Genetics Network. The polymorphisms that showed replication were further evaluated in subjects from the US National Emphysema Treatment Trial and controls from the Normative Aging Study and then in a fourth cohort of extended pedigrees from the Boston Early-Onset COPD population. Two polymorphisms in the α-nicotinic acetylcholine receptor 3/5 locus on chromosome 15 showed unambiguous evidence of association with COPD. This locus has previously been implicated in both smoking behavior and risk of lung cancer, suggesting the possibility of multiple functional polymorphisms in the region or a single polymorphism with wide phenotypic consequences. The hedgehog interacting protein (HHIP) locus on chromosome 4, which is associated with COPD, is also a significant risk locus for COPD.

## Introduction

COPD is expected to be the third leading cause of worldwide mortality and the fifth leading cause of morbidity by the year 2020 [1]. Cigarette smoking is the major risk factor for COPD but smokers show considerable variation in their risk of developing airflow obstruction. Familial aggregation studies suggest a strong genetic component to this risk [2]–[8]. However the only proven genetic risk factor for COPD is severe deficiency of α1-antitrypsin [9], which is present in only 1–2% of individuals with COPD. This suggests that other genes have yet to be identified that predispose smokers to airflow obstruction. We report the first genome wide association study (GWAS) for COPD. Our primary discovery sample was a case-control population collected from Bergen, Norway, and we used three independent study cohorts to further evaluate the top associations emerging from the GWAS analysis.

## Results

Baseline characteristics of the subjects used in the GWAS and subsequent replication studies are presented in Table 1.

**Table 1:** Characteristics of the primary screening and replication populations.

	COPD Case-Control Data		ICGN Family Data		NETT/NAS		Boston Early Onset COPD	
	Cases	Controls	Probands	Siblings	NETT Cases	NAS Controls	Probands	Relatives
Subjects	823	810	606	1285	389	472	127	822
Age (±SD)	65.41 (±10.15)	55.45 (±9.58)	58.43 (±5.40)	58.02 (±9.78)	67.44 (±5.82)	69.79 (±7.53)	48.09 (±4.70)	46.25 (±18.75)
Female (%)	330 (40.10%)	402 (49.63%)	244 (40.26%)	640 (49.80%)	139 (35.73%)	0	95 (74.80%)	458 (55.72%)
Post-FEV_1_ in liters (±SD)	1.59 (±0.71)	3.25 (±0.74)	1.11 (±0.44)	2.36 (±0.98)	0.81 (±0.26)	3.03 (±0.50) b	0.65 (±0.28) c	2.84 (±1.03) c
Post-FEV_1_, % pred (±SD)	50.26 (±17.33)	93.91 (±9.22)	36.19 (±12.94)	77.46 (±25.92)	28.00 (±7.36)	99.96(±13.12) b	21.86 (±8.44) c	87.22 (±20.27) c
Post-FEV_1_/FVC ratio (±SD) a	0.52 (±0.13)	0.79 (±0.04)	0.37 (±0.12)	0.61 (±0.15)	0.32 (±0.06)	0.79 (±0.05) b	30.88(±10.09) c	73.44 (±12.85) c
Pack-years of smoking (±SD)	31.83 (±18.86)	19.40(±13.43)	51.59 (±26.71)	40.49 (±24.62)	66.36(±30.37)	40.30 (±27.56)	38.86 (±21.87)	18..96 (±25.04)
Current smoking status (%)	383 (46.54%)	342 (42.22%)	205 (33.83%)	653 (50.82%)	0	31 (6.57%)	16 (12.60%)	248 (30.17%)

a Note: FEV_1_/VC was used for the ICGN Family Data, with VC determined by the higher of FVC and SVC.

b Pre-bronchodilator spirometry measurements were used in the NAS.

c Post-bronchodilator spirometry measurements available in 118 probands and 789 relatives.

### Genome-Wide Association Results

**Figure 1:**
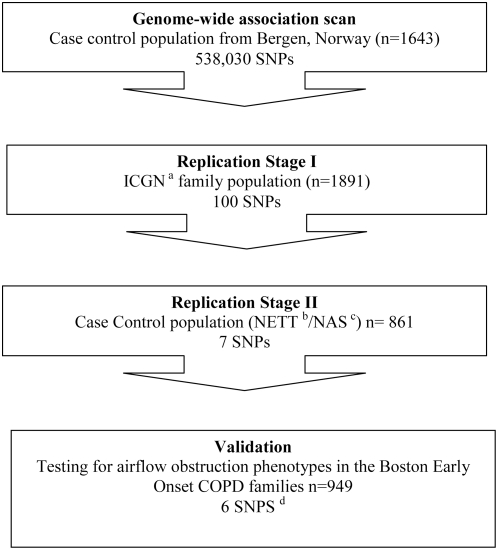
Study design. SNP: Single Nucleotide Polymorphism. ^a^ICGN: International COPD Genetics Network families. ^b^NETT: National Emphysema Treatment Trial. ^c^NAS: Normative aging study. ^d^ One of the SNPs genotyped in NETT/NAS population (rs735243) was not successfully genotyped in the BEOCOPD population.

We used a multi-stage replication design (Figure 1) for this study. The genome-wide association analyses of the COPD case-control status in the Bergen cohort identified several significant associations, including three SNPs on chromosome 5 that reached the level of genome-wide significance (Table S1). The Q-Q plot showing the distribution of observed P values from the discovery cohort is shown in online Figure S1. The top 100 SNPs were then evaluated in the ICGN population and 8 were replicated at a nominal p value of 0.05 (SNP rs11219732 showed inconsistent risk alleles in the Bergen and ICGN population and hence was not considered further, Table 2). Two of the three SNPs (rs7727670 and rs7341022 on chromosome 5) that showed genome-wide significance in the Bergen cohort did not replicate in the ICGN population. The SNPs showing the most definitive evidence for replication were rs8034191 and rs1051730 in the CHRNA3/5 locus.

**Table 2:** Results of the replication association analyses of the top 100 SNPs in the Norway, ICGN, and NETT/NAS populations.

Chromosome	SNP id	Bergen Case Control Population a			ICGN Population^b^		NETT/NAS c			Combined P value d	Gene/Nearest Gene
		Odds Ratio	Risk Allele	P value	p value (PBAT)	Risk Allele	Odds Ratio	P value	Risk Allele		
15	rs8034191	1.404	C	0.0001	2.21×10^−5^	C	1.43	0.0025	C	1.48×10^−10^	NP_001013641.2
15	rs1051730	1.39	T	0.0002	6.61×10^−6^	T	1.32	0.017	T	5.74×10^−10^	CHRNA3
5	rs9686327	1.847	A	9.8×10^−8^	0.0327	A	0.75	0.048	G e		ANKH
5	rs735243	1.893	T	2.02×10^−7^	0.0334	T	0.71	0.0348	C e		ANKH
11	rs11219732	0.35	C	4.47×10^−6^	0.0131	T e					CNTN5
2	rs6720264	1.452	A	6.69×10^−5^	0.0148	A	1.03	0.82	A	1.22×10^−5^	ACVR1
4	rs1828591	0.726	A	0.00016	0.0245	A	0.7	0.0019	A	1.47×10^−7^	HHIP
4	rs13118928	0.726	A	0.00016	0.0297	A	0.7	0.0018	A	1.67×10^−7^	HHIP

a Logistic regression analyses using age, gender, pack years of smoking and current smoking status as co-variates.

b International COPD Genetics Network (ICGN) population. PBAT version 3.6 analyses using age, gender, pack-years of smoking, current smoking status and center as co-variates.

c National Emphysema Treatment Trail/Normative aging study (NETT/NAS). Logistic regression using age and pack years of smoking (all the subjects are males and ex-smokers, so adjustments for gender and current smoking status were not included).

d Fisher's combined probability test was applied to combine the P-values from Bergen Cohort, ICGN cohort and NETT/NAS study.

P values in bold are above the genome-wide significance level (p<1.01×10^−7^).

Numbers in Bold are genome-wide significant (p value<1.01×10^−7^).

e Risk alleles were different in the ICGN or NETT/NAS population compared to the Bergen discovery cohort.

Several additional SNPs were later analyzed in the *CHRNA3/5* region in the Bergen and ICGN populations (Table S2). One non-synonymous polymorphism in *CHRNA5* (rs16969968) which coded for the substitution of an asparagine for an aspartic acid at amino acid 398) was associated with COPD in the Bergen (p = 8.8×10^−4^) and ICGN (p = 2.78×10^−6^) cohorts (combined p value 5.08×10^−8^). Since this SNP showed a weaker association than both rs8034191 and rs1051730 it was not considered as a causal variant.

We then tested the 7 SNPs that showed definite or nominal significance in the NETT-NAS case-control population, and the results are provided in Table 2. These results further confirmed the association of two SNPs at the CHRNA3/5 locus with COPD (p = 2.5×10^−3^, OR = 1.43, combined p value: 1.48×10^−10^ for rs8034191 and p = 0.017, OR = 1.32, combined p value 5.74×10^−10^ for rs1051730). Two SNPs (rs1828591 and rs13118928) at the HHIP locus on chromosome 4 also showed consistent replication across the three cohorts, but the combined p values did not reach genome-wide significance (1.47×10^−7^ and 1.67×10^−7^ respectively).

The only significant associations in the Boston Early-Onset COPD families were with the rs8034191 and rs1051730 SNPs at the CHRNA 3/5 locus (p = 0.03 and 0.03 respectively) and the rs1828591 and rs13118928 SNPs at the HHIP locus (p = 0.0025 and 0.0014 respectively) with post bronchodilator FEV_1_. None of the SNPs was significantly associated with a diagnosis of COPD. Since the ICGN cohort had recruited subjects with a wide range of lung function, we also analyzed the association of the CHRNA 3/5 markers with post bronchodilator FEV_1_ after adjusting for age, height, gender, pack years and smoking status. The results show that CHRNA 3/5 SNPs were associated with FEV_1_ in the ICGN population (p values 1.04×10^−4^ and 1.75×10^−5^ for rs8034191 and rs1051730 respectively).

**Figure 2:**
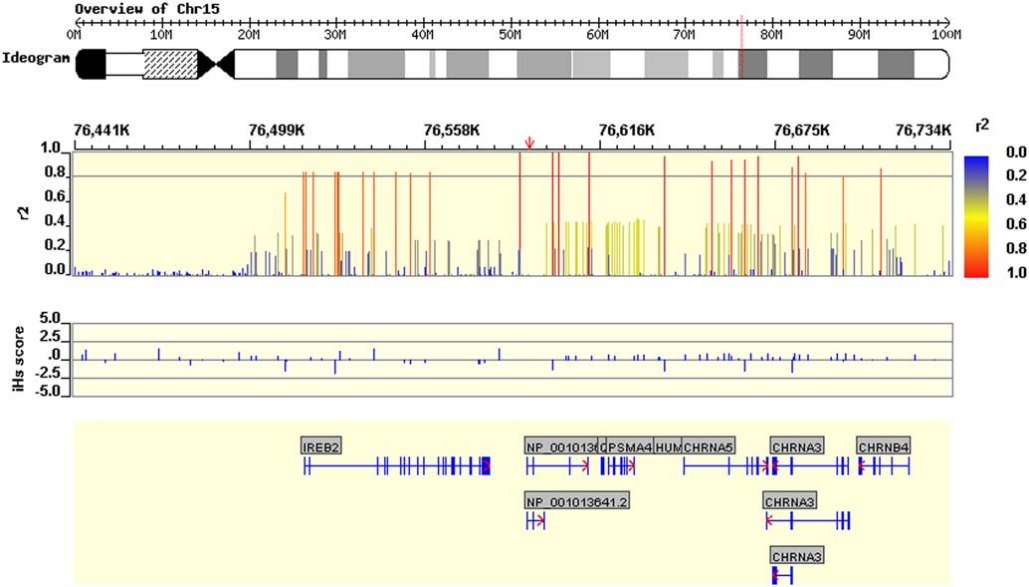
Region of Association around CHRNA3/CHRNA5. Annotated sections: Top: Linkage disequilibrium (r^2^) for all HapMap SNPs with rs8034191, showing an LD extension covering CHRNA3/CHRNA5. [red] r^2^≥0.8, [yellow] 0.5≤r2<0.8, [gray] 0.3≤r^2^<0.5, [blue] 0.2≤r2<0.3. Middle: Recent selection score. Bottom: genic context. Exons are depicted as blue vertical lines/rectangles, while introns are depicted as blue horizontal lines. Annotations were done using the WGAViewer software [31].

The COPD associated region on chromosome 15 spans seven genes (Figure 2). Cholinergic nicotinic receptor subtypes α3, α5 and β4; *IREB2*, *PSMA4*, *NP_001013641.2* (a gene with unknown function) and *Q9UD29* (Surfactant protein B (SP-B)-binding protein). A partial map of the region is shown in online Figure S2. SP-B binding protein is a DNA binding protein which binds to the promoter of SP-B and affects its expression [10]. Since SP-B is a critical surfactant in the lungs [11], we sequenced the SP-B binding protein in 30 COPD subjects who are homozygous for the risk allele of rs8034191 but did not identify any polymorphisms in this gene.

### Interaction with Smoking

The p values reported above were based on the adjusted analyses correcting for smoking exposure. The results from the unadjusted association analyses for COPD status were highly significant (Bergen 2×10^−4^ and 4×10^−4^; ICGN 7.46×10^−7^ and 1.40×10^−6^; NETT/NAS, 2.0×10^−5^ and 2.5×10^−4^ and combined p values of 1.86×10^−12^ and 6.6×10^−11^ for rs8034191 and rs1051730 respectively; Table S3). Although the adjustments for smoking exposure attenuated the p values, they still remained highly significant (Table 2). In the Norwegian discovery cohort, a significant genotype-by-environment interaction (P = 0.002, Table 3) was observed with a substantially higher risk of COPD in current smokers carrying the rs8034191 C allele (OR = 2.00) than in former smokers (OR = 1.10). In the overall population, the C allele of rs8034191 was estimated to have a population attributable risk of 12.2% for COPD. This risk was 14.3% in current smokers and 3.1% in former smokers. The p values were attenuated in the ICGN family-based population following adjustment for age, sex, pack-years of smoking and center but remained highly significant (Table 2). We identified a SNP by pack-years interaction (p = 0.0037 for rs8034191), however no significant SNP by current smoking status interaction (p = 0.85) was detected in the ICGN population.

**Table 3:** Genotype counts and allele frequencies for rs8034191 by case-control status and smoking status in the Bergen discovery cohort.

Population	Smoking Status/Amount	Genotype			MAF (C)
		TT	CT	CC	
case	All	326	375	122	0.376
ctrl	All	391	328	91	0.315
case	Current Smoker	139	186	58	0.394
case	Current Non-smoker	186	188	64	0.361
ctrl	Current Smoker	177	135	30	0.285
ctrl	Current Non-smoker	213	193	61	0.337
case	Heavy Smoker ^Note 1^	221	251	85	0.378
case	Light Smoker	105	124	37	0.372
ctrl	Heavy Smoker	128	115	34	0.330
ctrl	Light Smoker	263	213	57	0.307
case	Heavy Smoker ^Note 2^	233	247	79	0.362
case	Light Smoker	92	127	43	0.406
ctrl	Heavy Smoker	122	105	28	0.316
ctrl	Light Smoker	268	223	63	0.315
Norwegian population controls	Population sample	246	251	54	0.326

Note 1: Heavy smoker defined by pack-years of smoking (grouped by log transformed median = 3.11).

Note 2: Heavy smoker defined by length of smoking history (grouped by median = 41.5 yr).

Testing directly for an association between the amount of smoking, measured as pack-years, within cases and controls respectively, with the SNP rs8034191, demonstrated no such association in the Norway discovery cohort (P = 0.63 and 0.47, respectively). We also carried out tests comparing allele frequencies for current and former smokers and heavy and light smokers, (two different definitions, using pack years of smoking and length of smoking history) within cases and controls separately (Table 3). The only significant association observed was in comparing current and former smokers among the controls (p = 0.028). Similarly, the rs8034191 SNP was not associated with pack-years smoked in the NETT cases or in the NAS controls.

## Discussion

We have demonstrated and replicated genetic associations between SNPs at the CHRNA3/5 locus and COPD in four study populations. The estimated population attributable risk from this locus was 12.2% and represents the discovery of a common major locus contributing to COPD in the general population. However, a potential complication with the interpretation of these findings is the possibility that differences in smoking behavior, likely related to nicotine addiction, between COPD cases and controls may drive the observed association. This is similar to the recently reported association of CHRNA3/5 SNPs with lung cancer [12]–[14].

In the current study populations, only limited assessment of nicotine addiction is available: (i) whether subjects were still smoking at the time of study participation, and (ii) their lifetime smoking intensity. Thus, we have limited ability to disentangle a genetic determinant of smoking behavior from a genetic determinant of COPD through an alternative pathway. There are several pieces of evidences to suggest that there could be a direct effect of CHRNA3/5 locus on COPD, independent of smoking behavior. First, to the extent that smoking behavior is captured in pack-years, this effect should be factored out by the statistical design in which the discovery analyses used a logistic regression model incorporating pack-years, age and gender as covariates. The adjustments for pack-years smoked, age and gender were also performed in all the replication analyses. However, pack-years smoked only partially capture smoking behavior. Many other factors, such as depth of inhalation, number of puffs per cigarette, and age of starting smoking are also likely to affect the toxicant exposure and effect. Second, we also tested directly for an association between the amount of smoking, measured as pack-years, within cases and controls with the SNP rs8034191. There was no significant association between the SNPs and pack-years of smoking in the Bergen and NETT/NAS populations. This is consistent with the observed allele frequency among the Norwegian pediatric general population sample (minor allele frequency = 0.326, n = 551) which is between that observed for cases and controls and not significantly different from either.

We observed a genotype-by-environment interaction between the risk of the rs8034191 genotype and current smoking status on COPD in the Norwegian sample (P = 0.002, Table 3), showing a substantially higher risk of COPD in current smokers carrying the rs8034191 C allele (OR = 2.0) than in former smokers (OR = 1.1). There are several possible explanations for this gene-by-environment interaction. First, it could relate to nicotine addiction; smokers that have greater difficulty quitting smoking may be more likely to develop COPD. Alternatively, it could indicate that a subset of individuals is at greater risk for developing COPD if they continue to smoke. A similar interaction with current smoking was not identified in the ICGN families. Since all the COPD patients in the NETT population were former smokers, we could not address this question in the NETT/NAS study.

The association of smoking dependence was explored in the lung cancer report by Hung and colleagues [13] who did not detect any association with individual Fagerstrom indices of nicotine addiction or when comparing controls with a heaviness of smoking index (HSI). Another lung cancer report by Amos and colleagues [12] did show weak evidence for association with smoking behavior, while a further report by Thorgeirsson and colleagues[14] showed very strong association with smoking behavior. Association of this locus with nicotine dependence has been reported in two other studies [15],[16]. Thus, it is reasonable to conclude that the variants may affect smoking behavior, at the same time as having a significant effect on COPD and other smoking related diseases such as lung cancer and peripheral arterial disease [12]–[14].

The CHRNA3/5 SNPs were also associated with lung function (FEV_1_) in the ICGN and BEOCOPD populations. These SNPs were shown to be associated with FEV_1_ in the British Birth Cohort (rs8034191 and rs1051730, p = 0.029 and 0.023, respectively (http://www.b58cgene.sgul.ac.uk/, accessed [3/7/2008]). Historically, nicotinic receptors are classified as neuronal or muscle-type, based on their initial site of identification and composite subunits [17]. Cholinergic activity in the airways primarily induces tracheo-bronchial smooth muscle contraction and mucous secretion. However, there is an increasing body of literature showing the importance of extra-neuronal cholinergic signaling [18] in the lung.

The association of the SNPs at the chromosome 4 HHIP (Hedgehog-Interacting Protein) locus is also interesting, though it did not reach the stringent genome-wide significance levels in the populations studied in this manuscript. These SNPs were also associated with FEV_1_ in the BEOCOPD study (rs1828591 and rs13118928, p = 0.0025 and 0.0014). The same SNPs are also associated with FEV_1_ in the British Birth Cohort (rs1828591 and rs13118928, p = 0.039 and 0.038, respectively) but were not associated with FEV_1_ in the ICGN population.

In another manuscript in this issue of the journal, genome-wide association analysis results for FEV_1_/FVC in the Framingham Heart Study (FHS) are reported (Wilk et al). Due to differences in genotyping platforms, the most significantly associated SNPs on chromosome 15 in our study were not genotyped in FHS. Analysis of the genotyped SNPs in the chromosome 15 region in the FHS indicated no significant association with COPD, but association with FEV_1_ percent predicted was observed with one SNP in LD with rs8034191 (rs11636431 p value 0.007). Evaluation of the imputed data for the most significantly associated SNPs in our populations did not show association with COPD in FHS. Several factors could contribute to the absence of association to the COPD phenotype in FHS: (1) The FHS cohort is a population-based collection, while our studies evaluated populations ascertained specifically for COPD; (2) The FHS cohort was recruited over three decades, while our cohorts represent more recent recruitments (in the last 5–10 years)-smoking habits have changed over time, and it is also possible that COPD clinical characteristics have changed over this period; (3) Our cohorts include a greater proportion of severe COPD subjects than in FHS; and (4) There could be differences in linkage disequilibrium patterns between study populations. Further studies will be required to define the specific genetic determinants influencing COPD on chromosome 15, the relationship of these genetic factors to smoking behavior, and the characteristics of COPD subjects influenced by these genetic determinants.

The association of the Chromosome 4 region in the FHS cohort was genome-wide significant for the FEV_1_/FVC ratio and was also associated with COPD. This association was subsequently replicated in the Family Heart Study population. Though the HHIP locus association in our study did not reach genome-wide significance, the additional evidence from the FHS and Family Heart Study underscore the importance of this locus on COPD susceptibility.

We used independent populations with varying COPD severity, independent genotyping platforms and stringent statistical significance criteria to define genome-wide significant associations. We used consensus criteria for replication using a multi-stage replication design with similar phenotypes, the same genetic model and direction of association [19]. The levels of statistical significance of the association for our most significant results in the CHRNA3/5 region were consistent in all of the populations studied and are unlikely to be false positive results. The p values after adjusting for multiple testing using the most conservative Bonferroni correction were 7.3×10^−5^ and 2.83×10^−4^ for the SNPs rs8034191 and rs1051730 respectively. Though this can be considered as strength, the conservative approach for SNP confirmation that we have used may lead to larger false negative rates. However, with the inconsistent results of previous complex disease genetic association studies, we contend that a conservative approach is appropriate. We selected only the top 100 SNPs from the GWAS for subsequent replication study and a larger number of significant associations may have been uncovered if more of the most promising SNPs had been followed up. A negative association in the replication studies may not rule out a true association, since the power to detect association in the replication populations may be limited. The primary replication cohort (ICGN) is moderately powered to detect the replicated associations. Though the sample sizes of the NETT/NAS and BEOCOPD studies are relatively low, these studies include a large percentage of severely affected individuals, who may be enriched for COPD susceptibility genes. This likely account for the high rate of replication in these populations. COPD is a heterogeneous disease and we used a spirometry-based definition for COPD in all of the populations. Differences in smoking exposure, current smoking status, entry criteria and geographic origin of the cohorts might contribute to phenotypic heterogeneity and may lead to lack of replication. The fact that the replicated associations holds-up strongly and consistently in all the populations shows that phenotypic heterogeneity likely has little effect on the most significant results.

In summary, we have identified robust evidence of association of COPD with the α-nicotinic receptor (CHRNA 3/5) and HHIP loci. The hedgehog (Hh) gene family encodes signaling molecules that play an important role in regulating morphogenesis and the HHIP locus may play a role lung development. Although there is evidence of association of CHRNA 3/5 locus with nicotine addiction, both this study and recent reports of a lung cancer association [12]–[14] with the same alleles suggest that this region may be involved in more than nicotine addiction, and may potentially have direct functional relevance in the development of COPD, lung cancer, peripheral arterial disease, and other smoking related conditions. The first-degree relatives of both lung-cancer patients and COPD patients have higher rates of impaired forced expiratory flow rates than relatives of patients with non-pulmonary disease [20], suggesting that susceptibility to lung cancer and COPD share common familial components. The association of *CHRNA 3/5* locus with COPD, lung cancer, and peripheral arterial disease is powerful enough to make genetic screening of smokers an attractive interventional strategy.

## Materials and Methods

### Study Participants and Phenotypes

Subjects from a case-control study [21],[22] recruited from Bergen, Norway were used as the discovery cohort in the GWAS. Baseline characteristics of the subjects are shown in Table 1. The entry criteria for COPD cases were post-bronchodilator forced expiratory volume in 1 second (FEV_1_) <80% predicted and FEV_1_/FVC (forced vital capacity) <0.7. The controls were selected based on post-bronchodilator FEV_1_ >80% predicted and FEV_1_/FVC >0.7. Individuals with Pi ZZ, ZNull, Null-Null or SZ α_1_-antitrypsin deficiency were excluded. Subjects with chronic pulmonary disorders other than COPD (e.g., lung cancer, sarcoidosis, active tuberculosis, and lung fibrosis) were also excluded. Because of the potential overlap in susceptibility genes for COPD and asthma, and the difficulty of diagnosing COPD vs. asthma in smokers with chronic airflow obstruction, previous asthma diagnosis was not used as an exclusion criterion. Both cases and controls were required to have a minimum of 2.5 pack-years of smoking. A total of 823 COPD cases and 810 controls were included in the present analysis. All of the subjects used in the primary and replication populations were current or former smokers (Table 1). Although the mean number of pack-years smoked was higher in cases (mean 32 SD 18) compared with controls (mean 19 SD 13), subjects with a range of smoking intensities were included in the analysis. The distribution of pack-years of smoking in cases and controls in the Bergen cohort is shown in Figure S3.

Subjects from the International COPD Genetics Network (ICGN) were used as the primary replication population. In the multi-center ICGN study [22],[23] subjects with known COPD were recruited as probands, and siblings and available parents were ascertained through the probands. Inclusion criteria for probands were post-bronchodilator FEV_1_<60% predicted and FEV_1_/VC <90% predicted at a relatively early age (45 to 65 years), a≥5 pack-year smoking history, and at least one eligible sibling with a≥5 pack-year smoking history. COPD in siblings was defined by a post-bronchodilator FEV_1_<80% predicted and FEV_1_/VC<90% predicted. The same exclusion criteria used in the Bergen study were also applied for the ICGN population. In total, 1891 Caucasian individuals from 606 pedigrees were included in the ICGN family-based association analysis.

The second replication cohort included 389 non-Hispanic white COPD cases from the U.S. National Emphysema Treatment Trial (NETT) [24] and 472 non-Hispanic white control subjects from the Normative Aging Study (NAS) [25]. Subjects in NETT had severe COPD (FEV_1_ ≤45% predicted) and bilateral emphysema on chest CT; all NETT subjects were former smokers. Control subjects from the NAS had normal spirometry and at least 10 pack-years of cigarette smoking history. Subjects from extended pedigrees in the Boston Early-Onset COPD (BEOCOPD) study were used as an additional family-based replication cohort. BEOCOPD subjects were recruited through COPD probands with age <53 years, FEV_1_ <40% predicted, and without severe α_1_-antitrypsin deficiency [26]. The BEOCOPD analysis included 949 individuals from 127 pedigrees.

Finally, to estimate allele frequencies in the general population in Norway we used 551 children (all non-smoking) from the Environment and Childhood Asthma (ECA) birth cohort study in Oslo [27].

All participants provided written informed consent and local institutional review boards approved the study protocols.

### Genotyping and Quality Control

All samples in the Bergen discovery cohort were genotyped using Illumina's HumanHap550 genotyping BeadChip (version 3) which contains 561,466 single nucleotide polymorphisms (SNPs). All samples that had a call rate <98%, and all SNPs that had a call frequency <99% were deleted. This resulted in the elimination of 23,436 SNPs from further analysis (See Text S1 for more details). The ICGN subjects were genotyped using Sequenom's iPLEX SNP genotyping protocol developed for measurement with the MassARRAY mass spectrometer [28]. Genotyping in the NETT/NAS and BEOCOPD studies was performed using Sequenom iPLEX or Applied Biosystems TaqMan assays. Genotyping in the Norwegian ECA Birth cohort was done by TaqMan.

### Statistical Analysis

For the association analyses COPD affection status in the Norway discovery cohort, we used a logistic regression model to perform single-marker genotype trend tests for the QC-passed SNPs. To control for the possibility of spurious associations resulting from population stratification, we used a modified EIGENSTRAT method [29] (and Text S1). This showed that there were 12 significant principal component axes, all of which were included in the model. We included age and sex, and since smoking effects are known to influence COPD risk, we also included current smoking status and pack-years of smoking as co-variates.

The top 100 SNPs showing the lowest P values in this stage were selected for assessment in replication cohorts. For replication, we used a two stage strategy using three independent cohorts (Figure 1). In the first stage, family-based association analysis for COPD affection status was conducted in the ICGN data using PBAT version 3.6 [30]. Adjustments for age, gender, pack-years of smoking, current smoking status and center were performed in order to take into account the effect of smoking on the association results. Association with FEV_1_ was also tested using PBAT with age, gender, pack-years of smoking, current smoking status and height as co-variates. Gene-by-environment interaction analyses were also conducted using the PBAT program. Biallelic tests were conducted for SNPs using an additive genetic model. In the second stage the NETT case-control study was analyzed for the presence/absence of COPD using an additive genetic model. An unadjusted analysis and a logistic regression model adjusted for age and pack-years of smoking were conducted; sex was not included as a covariate because all NAS subjects were male, and current smoking was not included because all NETT subjects were ex-smokers. The BEOCOPD family-based study in the validation stage was analyzed using PBAT version 3.6 [30]. COPD was defined by post-bronchodilator FEV_1_/FVC<0.7 and FEV_1_<80% predicted (GOLD stage 2 or greater). Because a broad range of FEV_1_ values were included in the BEOCOPD study, we also analyzed FEV_1_ as a quantitative outcome in that population. Analysis of post-bronchodilator values of FEV_1_ was adjusted for ever-smoking status, pack-years of smoking, age, sex, and height.

### Adjustments for Multiple Comparisons

We assessed genome-wide significance with a Bonferroni correction (p cutoff = 1.013×10^−7^ considering 493,609 independent tests in the Bergen cohort (see Text S1), 100 tests in the ICGN cohort, 7 tests in the NETT/NAS study and 6 tests in the BEOCOPD study (Total 493,772 tests).

## Supporting Information

Figure S1 Q-Q plot showing the distribution of observed P values.
(0.03 MB DOC)

Figure S2 Partial map of the *CHRNA5/CHRNA3* region.
(0.10 MB DOC)

Figure S3 Distribution of pack years of smoking in cases and controls from the Bergen, Norway cohort.
(0.02 MB DOC)

Table S1 Results of the genome-wide association analysis from the Bergen cohort.
(0.18 MB DOC)

Table S2 Results of the association analysis using additional SNPs in the CHRNA3/5 region in the Bergen cohort and the ICGN study.
(0.14 MB DOC)

Table S3 Results of the unadjusted analyses of the Bergen, ICGN and NETT/NAS populations.
(0.04 MB DOC)

Text S1 Supplementary materials.
(0.05 MB DOC)
