# Supplementary material for: A Genome-Wide Association Study in Chronic Obstructive Pulmonary Disease (COPD): Identification of Two Major Susceptibility Loci
Source: PLoS Genet. 2009 Mar 20;5(3):e1000421. doi: 10.1371/journal.pgen.1000421 (PMC2650282; doi:10.1371/journal.pgen.1000421)
Supplement: Figure S1 — Q-Q plot showing the distribution of observed P values. (0.03 MB DOC) [file pgen.1000421.s001.doc]

**Online Supplementary Figure 1**


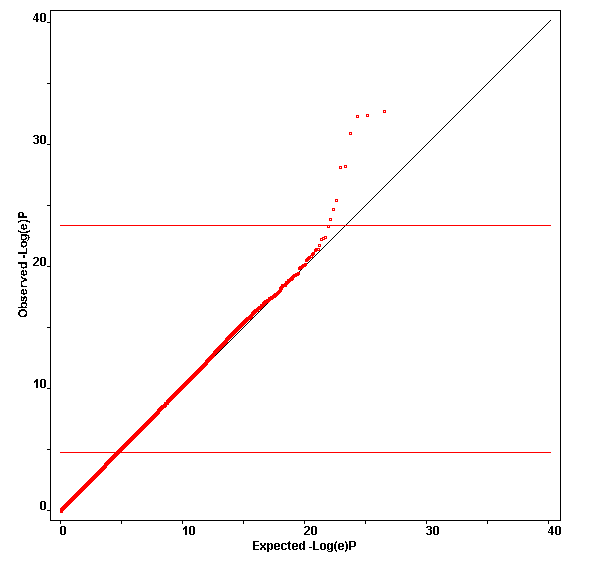


Q-Q plot showing the distribution of observed P values. Displayed included 15 data points lifting above the expected distribution, and an estimated inflation factor (λ) of 1.023, indicating no significant overall effect of stratification (and indicating that the method used here is sufficient to control for stratification).
