# Supplementary material for: A Genome-Wide Association Study in Chronic Obstructive Pulmonary Disease (COPD): Identification of Two Major Susceptibility Loci
Source: PLoS Genet. 2009 Mar 20;5(3):e1000421. doi: 10.1371/journal.pgen.1000421 (PMC2650282; doi:10.1371/journal.pgen.1000421)
Supplement: Figure S2 — Partial map of the CHRNA5/CHRNA3 region. (0.10 MB DOC) [file pgen.1000421.s002.doc]

Online Supplementary Figure 2


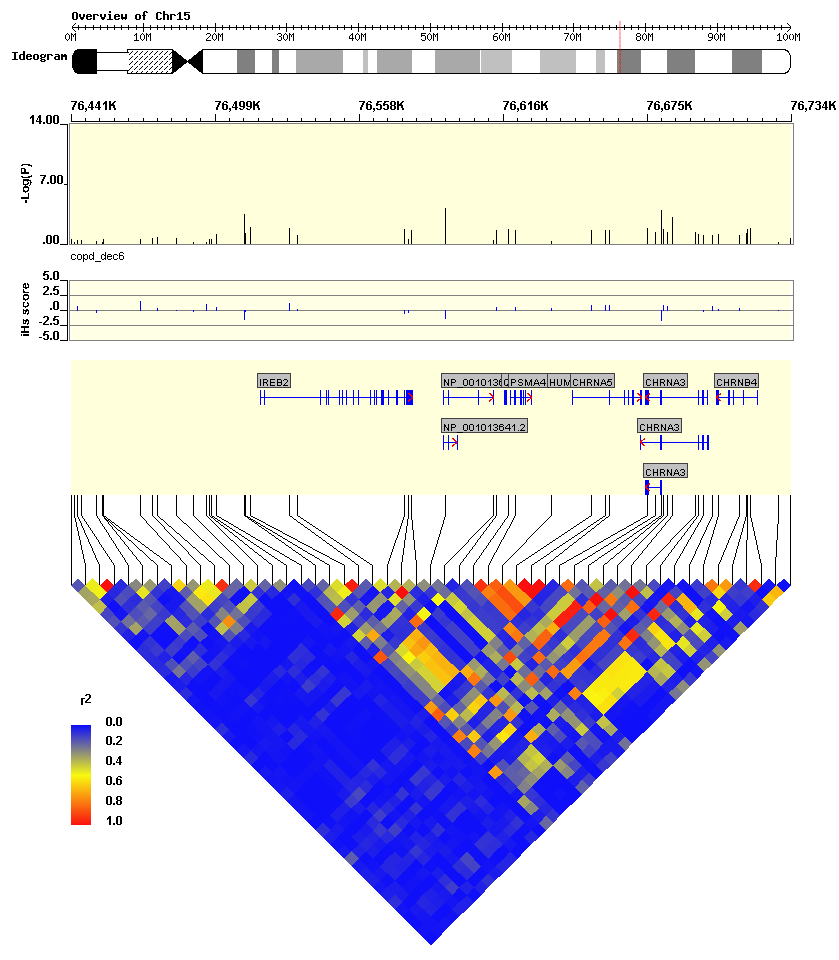


Partial map of the *CHRNA5/CHRNA3* region. Indicated are the *P*-values [-log(P)] of all genotyped SNPs annotated with the gene structure. Annotated sections: Top: -log(P) for all tested SNPs in this region; Middle 1: Recent selection score. Middle 2: genic context. Exons are depicted as blue vertical lines/rectangles, while introns are depicted as blue horizontal lines. Bottom: Linkage disequilibrium (r^2^) for all tested SNPs, [red] r^2^ ≥ 0.8, [yellow] 0.5 ≤ r2 < 0.8, [gray] 0.3 ≤ r^2^ < 0.5, [blue] 0.2 ≤ r2 < 0.3. Annotations were done using the WGAViewer software.
