# Supplementary material for: A Genome-Wide Association Study in Chronic Obstructive Pulmonary Disease (COPD): Identification of Two Major Susceptibility Loci
Source: PLoS Genet. 2009 Mar 20;5(3):e1000421. doi: 10.1371/journal.pgen.1000421 (PMC2650282; doi:10.1371/journal.pgen.1000421)
Supplement: Figure S3 — Distribution of pack years of smoking in cases and controls from the Bergen, Norway cohort. (0.02 MB DOC) [file pgen.1000421.s003.doc]

Online **Supplementary Figure 3**


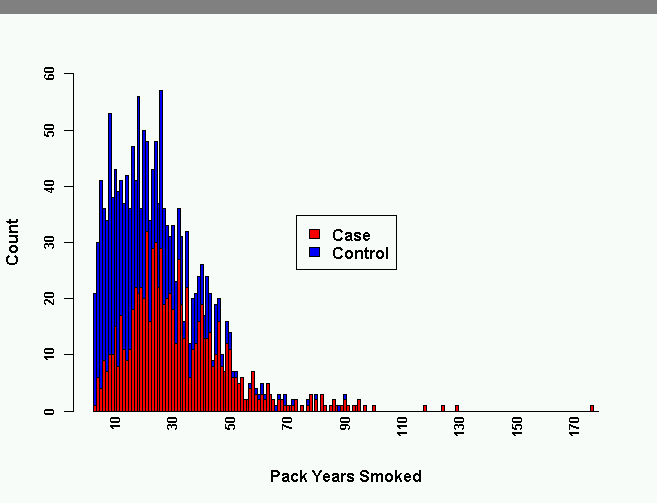


Distribution of pack years of smoking in cases and controls from the Bergen, Norway cohort. The graph is a stacked bar chart where the stacked bars sum to the total number of cases and control at a pack year range.
