# Supplementary material for: A Genome-Wide Association Study in Chronic Obstructive Pulmonary Disease (COPD): Identification of Two Major Susceptibility Loci
Source: PLoS Genet. 2009 Mar 20;5(3):e1000421. doi: 10.1371/journal.pgen.1000421 (PMC2650282; doi:10.1371/journal.pgen.1000421)
Supplement: Table S1 — Results of the genome-wide association analysis from the Bergen cohort. (0.18 MB DOC) [file pgen.1000421.s004.doc]

**Supplementary Table 1.** Results of the genome-wide association analysis from the Bergen cohort. P values and Odds ratios from the top 100 SNPs

| Chromosome | Ref SNP id | Location NCBI 36 | Odds ratio | | | Risk Allele | P-value |
| --- | --- | --- | --- | --- | --- | --- | --- |
|  |  |  | OR | Lower 95 | Upper 95 |  |  |
| 5 | rs7727670 | 15075112 | 1.863 | 1.484 | 2.34 | T | 8.38E-08 |
| 5 | rs9686327 | 15071537 | 1.847 | 1.474 | 2.315 | A | 9.84E-08 |
| 5 | rs7341022 | 15067182 | 1.845 | 1.473 | 2.311 | A | 9.97E-08 |
| 5 | rs735243 | 15092327 | 1.893 | 1.488 | 2.409 | T | 2.02E-07 |
| 5 | rs1080879 | 15107802 | 1.733 | 1.394 | 2.155 | C | 7.69E-07 |
| 5 | rs32466 | 15140791 | 1.745 | 1.398 | 2.177 | T | 8.31E-07 |
| 5 | rs30539 | 15097842 | 1.648 | 1.335 | 2.034 | T | 3.38E-06 |
| 11 | rs11219732 | 98763035 | 0.3501 | 0.2236 | 0.5481 | C | 4.47E-06 |
| 1 | rs7524799 | 164202205 | 0.6801 | 0.575 | 0.8044 | T | 6.74E-06 |
| 1 | rs11588172 | 25294577 | 1.476 | 1.243 | 1.754 | T | 9.02E-06 |
| 1 | rs7522756 | 164228562 | 1.467 | 1.234 | 1.744 | A | 1.44E-05 |
| 6 | rs1575208 | 72403193 | 1.575 | 1.283 | 1.935 | T | 1.46E-05 |
| 3 | rs9862661 | 56045798 | 1.48 | 1.239 | 1.769 | C | 1.57E-05 |
| 1 | rs6426962 | 164262955 | 1.461 | 1.228 | 1.738 | A | 1.94E-05 |
| 6 | rs12193019 | 72357255 | 1.511 | 1.248 | 1.829 | C | 2.27E-05 |
| 5 | rs16903825 | 15167063 | 3.22 | 1.873 | 5.537 | C | 2.35E-05 |
| 2 | rs1551133 | 119253354 | 2.191 | 1.523 | 3.153 | A | 2.38E-05 |
| 3 | rs13061634 | 56029117 | 1.467 | 1.227 | 1.756 | C | 2.76E-05 |
| 3 | rs12495172 | 55968696 | 1.447 | 1.217 | 1.719 | A | 2.79E-05 |
| 16 | rs239349 | 23260349 | 1.463 | 1.223 | 1.749 | A | 3.09E-05 |
| 6 | rs10942957 | 72393738 | 1.556 | 1.264 | 1.916 | T | 3.15E-05 |
| 1 | rs215864 | 37740361 | 0.5959 | 0.4667 | 0.7609 | A | 3.30E-05 |
| 11 | rs12421122 | 98659612 | 0.5219 | 0.3835 | 0.7102 | C | 3.52E-05 |
| 4 | rs6823107 | 24053883 | 1.471 | 1.225 | 1.766 | C | 3.62E-05 |
| 2 | rs4432437 | 59459080 | 1.584 | 1.271 | 1.974 | T | 4.22E-05 |
| 1 | rs11210569 | 38578516 | 0.7074 | 0.5992 | 0.8352 | T | 4.40E-05 |
| 8 | rs2589183 | 97591685 | 0.6272 | 0.5013 | 0.7847 | C | 4.50E-05 |
| 10 | rs12762979 | 28788830 | 1.562 | 1.26 | 1.935 | A | 4.59E-05 |
| 4 | rs1996020 | 145730644 | 1.641 | 1.292 | 2.083 | C | 4.86E-05 |
| 1 | rs732285 | 164234044 | 1.431 | 1.204 | 1.7 | C | 4.86E-05 |
| 11 | rs4754595 | 98595718 | 0.5531 | 0.4156 | 0.7361 | A | 4.90E-05 |
| 6 | rs7775523 | 21063311 | 0.6985 | 0.5872 | 0.831 | T | 5.12E-05 |
| 14 | rs1738899 | 96605676 | 2.073 | 1.452 | 2.961 | A | 6.09E-05 |
| 1 | rs7529406 | 79455123 | 1.591 | 1.268 | 1.996 | C | 6.13E-05 |
| 5 | rs25796 | 111472421 | 1.415 | 1.193 | 1.677 | G | 6.43E-05 |
| 5 | rs32447 | 15170438 | 1.414 | 1.193 | 1.675 | A | 6.46E-05 |
| 3 | rs17310770 | 125182513 | 0.6347 | 0.5077 | 0.7936 | A | 6.65E-05 |
| 4 | rs6852830 | 145726008 | 1.629 | 1.282 | 2.071 | A | 6.66E-05 |
| 2 | rs6720264 | 158499806 | 1.452 | 1.209 | 1.745 | A | 6.69E-05 |
| 14 | rs1622472 | 96599174 | 2.136 | 1.471 | 3.103 | C | 6.71E-05 |
| 14 | rs17099345 | 61470936 | 0.5271 | 0.3845 | 0.7226 | G | 6.93E-05 |
| 3 | rs11719713 | 136924121 | 0.5906 | 0.4553 | 0.766 | C | 7.22E-05 |
| 2 | rs935381 | 168649641 | 1.731 | 1.319 | 2.271 | T | 7.47E-05 |
| 22 | rs2269640 | 24667911 | 1.498 | 1.226 | 1.83 | C | 7.54E-05 |
| 11 | rs12576370 | 98696827 | 0.5381 | 0.3958 | 0.7314 | C | 7.59E-05 |
| 2 | rs4588237 | 47796406 | 0.616 | 0.4845 | 0.7832 | A | 7.66E-05 |
| 15 | rs16943236 | 84034355 | 0.3463 | 0.2047 | 0.5858 | C | 7.68E-05 |
| 18 | rs7233241 | 49347873 | 1.399 | 1.184 | 1.652 | T | 7.76E-05 |
| 5 | rs26566 | 111471653 | 0.6611 | 0.5384 | 0.8118 | A | 7.81E-05 |
| 14 | rs200303 | 98472186 | 1.418 | 1.192 | 1.686 | T | 7.96E-05 |
| 11 | rs12577504 | 98670876 | 0.5392 | 0.3965 | 0.7332 | C | 8.16E-05 |
| 11 | rs10769813 | 7598607 | 0.7087 | 0.5969 | 0.8415 | G | 8.54E-05 |
| 6 | rs4712564 | 21028102 | 0.6733 | 0.5525 | 0.8204 | A | 8.79E-05 |
| 6 | rs9350301 | 21048740 | 0.6805 | 0.5613 | 0.825 | T | 8.93E-05 |
| 2 | rs4148777 | 169578147 | 2.524 | 1.588 | 4.012 | C | 9.07E-05 |
| 1 | rs3767943 | 41066006 | 1.611 | 1.269 | 2.047 | A | 9.18E-05 |
| 14 | rs8009673 | 31412453 | 0.6067 | 0.472 | 0.7798 | C | 9.54E-05 |
| 3 | rs4974153 | 55944898 | 1.434 | 1.196 | 1.718 | T | 9.55E-05 |
| 1 | rs10926189 | 233827440 | 1.429 | 1.194 | 1.711 | T | 9.95E-05 |
| 5 | rs979455 | 150574995 | 0.701 | 0.5861 | 0.8386 | A | 0.00010 |
| 7 | rs9639549 | 27476118 | 0.7049 | 0.591 | 0.8409 | A | 0.00010 |
| 16 | rs3747581 | 4402620 | 1.465 | 1.208 | 1.776 | A | 0.00010 |
| 15 | rs8034191 | 76593078 | 1.404 | 1.183 | 1.666 | C | 0.00010 |
| 20 | rs4458268 | 43410405 | 1.411 | 1.186 | 1.68 | G | 0.00010 |
| 14 | rs17090892 | 55577553 | 0.5483 | 0.4046 | 0.7429 | A | 0.00010 |
| 12 | rs11177790 | 68364250 | 1.825 | 1.346 | 2.474 | A | 0.00010 |
| 9 | rs12339991 | 125550906 | 1.484 | 1.215 | 1.813 | A | 0.00011 |
| 5 | rs27536 | 111470610 | 0.6692 | 0.5458 | 0.8205 | A | 0.00011 |
| 10 | rs2488825 | 56759426 | 1.479 | 1.212 | 1.805 | A | 0.00011 |
| 1 | rs9426841 | 164234585 | 1.403 | 1.181 | 1.667 | A | 0.00011 |
| 19 | rs3787025 | 18283364 | 0.7181 | 0.6065 | 0.8503 | G | 0.00012 |
| 16 | rs875649 | 19571695 | 0.7162 | 0.6038 | 0.8495 | C | 0.00012 |
| 7 | rs12537593 | 66503610 | 1.683 | 1.289 | 2.196 | C | 0.00012 |
| 1 | rs12097924 | 71171490 | 0.6666 | 0.5416 | 0.8205 | G | 0.00013 |
| 4 | rs766908 | 185705879 | 1.622 | 1.266 | 2.077 | G | 0.00013 |
| 15 | rs1530169 | 72024173 | 1.475 | 1.208 | 1.801 | T | 0.00013 |
| 7 | rs10250721 | 130538334 | 0.5986 | 0.46 | 0.7791 | A | 0.00013 |
| 1 | rs3819783 | 71215214 | 0.6646 | 0.5385 | 0.8201 | T | 0.00014 |
| 11 | rs4938648 | 110840485 | 1.396 | 1.175 | 1.657 | T | 0.00014 |
| 5 | rs40394 | 111480276 | 1.454 | 1.199 | 1.763 | A | 0.00014 |
| 17 | rs1807333 | 895321 | 1.411 | 1.182 | 1.686 | T | 0.00014 |
| 1 | rs6540985 | 9631275 | 0.72 | 0.6079 | 0.8529 | A | 0.00014 |
| 16 | rs1548446 | 19599015 | 0.7182 | 0.6054 | 0.852 | C | 0.00014 |
| 3 | rs6789670 | 171648832 | 0.6524 | 0.5233 | 0.8133 | T | 0.00014 |
| 3 | rs11709483 | 56010925 | 1.425 | 1.187 | 1.71 | T | 0.00014 |
| 6 | rs4712572 | 21069040 | 0.6898 | 0.5694 | 0.8355 | G | 0.00014 |
| 4 | rs973852 | 97122688 | 0.7157 | 0.6021 | 0.8508 | A | 0.00014 |
| 6 | rs9368262 | 21102377 | 0.6893 | 0.5687 | 0.8355 | T | 0.00014 |
| 17 | rs8075200 | 24370680 | 1.415 | 1.182 | 1.694 | A | 0.00015 |
| 1 | rs4653305 | 37830453 | 0.618 | 0.4816 | 0.7931 | G | 0.00015 |
| 12 | rs1240265 | 68524759 | 1.381 | 1.168 | 1.633 | A | 0.00015 |
| 15 | rs1051730 | 76681394 | 1.39 | 1.172 | 1.649 | T | 0.00015 |
| 4 | rs13118928 | 145705839 | 0.7258 | 0.6145 | 0.8571 | A | 0.00015 |
| 4 | rs1828591 | 145700230 | 0.7258 | 0.6146 | 0.8572 | A | 0.00016 |
| 7 | rs12535529 | 66501385 | 1.659 | 1.275 | 2.159 | G | 0.00016 |
| 4 | rs4689860 | 7829563 | 0.6828 | 0.5599 | 0.8326 | T | 0.00016 |
| 5 | rs12152753 | 111470993 | 1.408 | 1.178 | 1.681 | A | 0.00016 |
| 5 | rs3734046 | 150881493 | 1.428 | 1.186 | 1.719 | G | 0.00016 |
| 16 | rs12934964 | 82332355 | 0.5431 | 0.3953 | 0.7462 | A | 0.00016 |
| 17 | rs8075175 | 26940757 | 1.433 | 1.188 | 1.728 | A | 0.00016 |
