# Supplementary material for: A Genome-Wide Association Study in Chronic Obstructive Pulmonary Disease (COPD): Identification of Two Major Susceptibility Loci
Source: PLoS Genet. 2009 Mar 20;5(3):e1000421. doi: 10.1371/journal.pgen.1000421 (PMC2650282; doi:10.1371/journal.pgen.1000421)
Supplement: Table S2 — Results of the association analysis using additional SNPs in the CHRNA3/5 region in the Bergen cohort and the ICGN study. (0.14 MB DOC) [file pgen.1000421.s005.doc]

**Supplementary Table 2.** Results of the association analysis using additional SNPs in the CHRNA3/5 region in the Bergen cohort and the ICGN study

|  |  |  | BERGEN COHORT | | | | | ICGN FAMILY DATA | | |
| --- | --- | --- | --- | --- | --- | --- | --- | --- | --- | --- |
| **Name** | **Gene** | NCBI 36 Location | ODDS RATIO | L95 | U95 | Risk Allele | P-value | Risk Allele | FBAT P value | PBAT P value |
| RS2568498 |  | 76508987 | 0.7992 | 0.6762 | 0.9445 | A | 0.008559 |  |  |  |
| RS1394371 |  | 76511524 | 1.348 | 1.129 | 1.611 | T | 0.0009898 |  |  |  |
| RS12903150 |  | 76511700 | 0.8387 | 0.698 | 1.008 | A | 0.06052 |  |  |  |
| RS17483548 | IREB2 | 76517368 | 1.405 | 1.179 | 1.673 | A | 0.0001397 |  |  |  |
| RS10519198 | IREB2 | 76529809 | 0.8205 | 0.694 | 0.9699 | C | 0.02045 |  |  |  |
| RS1964678 | IREB2 | 76541055 | 0.8472 | 0.7125 | 1.007 | C | 0.06051 |  |  |  |
| RS3817092 | IREB2 | 76551340 | 0.8629 | 0.6943 | 1.072 | T | 0.1838 |  |  |  |
| RS4299116 | IREB2 | 76553249 | 0.8297 | 0.698 | 0.9863 | A | 0.03428 |  |  |  |
| RS11634990 | IREB2 | 76558241 | 0.8849 | 0.7101 | 1.103 | T | 0.2761 |  |  |  |
| RS13180 | IREB2 | 76576543 | 0.8265 | 0.6955 | 0.9822 | T | 0.03048 |  |  |  |
| RS8034191 | LOC123688 | 76593078 | 1.394 | 1.173 | 1.656 | C | 0.0001584 | C | 7.86E-07 | 2.20966E-05 |
| RS10519203 | LOC123688 | 76601101 | 1.392 | 1.168 | 1.659 | G | 0.0002175 |  |  |  |
| RS12915366 | PSMA4 | 76618808 | 0.8332 | 0.705 | 0.9848 | G | 0.03241 |  |  |  |
| RS12916483 | PSMA4 | 76619452 | 0.8323 | 0.7041 | 0.984 | G | 0.0316 |  |  |  |
| RS3813572 | PSMA4 | 76619643 | 0.8242 | 0.697 | 0.9745 | A | 0.0237 |  |  |  |
| RS3813571 | PSMA4 | 76619847 | 0.8395 | 0.71 | 0.9927 | C | 0.04084 |  |  |  |
| RS3813570 | PSMA4 | 76619887 | 0.7827 | 0.6251 | 0.98 | A | 0.03269 |  |  |  |
| RS17554464 | PSMA4 | 76619887 | 0.8145 | 0.6532 | 1.016 | T | 0.06848 |  |  |  |
| RS4886571 | PSMA4 | 76620813 | 0.8593 | 0.7269 | 1.016 | A | 0.07568 |  |  |  |
| RS2292117 | PSMA4 | 76621744 | 0.8629 | 0.7301 | 1.02 | G | 0.08375 |  |  |  |
| RS4887062 | PSMA4 | 76624856 | 0.8585 | 0.7254 | 1.016 | A | 0.07599 |  |  |  |
| RS8053 | PSMA4 | 76628275 | 0.8376 | 0.7094 | 0.989 | C | 0.03662 |  |  |  |
| RS1979906 | PSMA4 | 76629344 | 0.8657 | 0.7313 | 1.025 | A | 0.09366 |  |  |  |
| RS4275821 |  | 76636596 | 0.8529 | 0.718 | 1.013 | T | 0.06995 | T | 0.002891 | 0.007292224 |
| RS2036527 |  | 76638670 | 1.388 | 1.17 | 1.647 | T | 0.0001689 | T | 3.91E-06 | 5.10728E-05 |
| RS667282 | CHRNA5 | 76650527 | 0.8138 | 0.6582 | 1.006 | T | 0.05714 | T | 0.011787 | 0.034425293 |
| RS6495306 | CHRNA5 | 76652948 | 0.8324 | 0.7048 | 0.983 | A | 0.03065 | A | 0.008427 | 0.004846222 |
| RS601079 | CHRNA5 | 76656634 | 0.8247 | 0.6988 | 0.9732 | T | 0.02255 | T | 0.005978 | 0.004256442 |
| RS621849 | CHRNA5 | 76659916 | 0.8553 | 0.7241 | 1.01 | A | 0.06576 | A | 0.016157 | 0.014614906 |
| RS16969968 | CHRNA5 | 76669980 | 1.384 | 1.143 | 1.677 | A | 0.0008767 | A | 2.78E-06 | 1.6437E-05 |
| RS1051730 | CHRNA3 | 76681394 | 1.39 | 1.172 | 1.649 | T | 0.0001578 | T | 1.40E-06 | 6.61431E-06 |
| RS1317286 | CHRNA3 | 76683184 | 1.395 | 1.175 | 1.655 | G | 0.0001392 | G | 1.57E-06 | 1.5801E-05 |
| RS3743075 | CHRNA3 | 76696507 | 0.8721 | 0.7366 | 1.032 | G | 0.1121 | G | 0.016324 | 0.012880673 |
| RS3743074 | CHRNA3 | 76696535 | 0.8804 | 0.7434 | 1.043 | T | 0.1397 | T | 0.017971 | 0.016505211 |
| RS8192475 | CHRNA3 | 76698285 | 0.7705 | 0.5467 | 1.086 | G | 0.1363 | G | 0.946545 | 0.947113961 |
| RS2067808 | CHRNA3 | 76698835 | 0.8924 | 0.7535 | 1.057 | C | 0.187 | C | 0.005282 | 0.006644143 |
| RS6495309 | CHRNA3, CHRNB4 | 76702300 | 0.8444 | 0.6778 | 1.052 | C | 0.1316 | C | 0.007425 | 0.019055605 |
| RS1948 | CHRNB4 | 76704454 | 0.8895 | 0.7498 | 1.055 | C | 0.179 | C | 0.130502 | 0.101726886 |
| RS7178270 | CHRNB4 | 76708132 | 0.838 | 0.7098 | 0.9893 | C | 0.03691 | C | 0.156541 | 0.176798842 |
| RS950776 | CHRNB4 | 76713073 | 0.9002 | 0.7595 | 1.067 | T | 0.2253 | T | 0.121171 | 0.155550993 |
| RS11637890 | CHRNB4 | 76722474 | 0.8579 | 0.7261 | 1.014 | C | 0.07183 | C | 0.141014 | 0.12010292 |
| RS3971872 |  | 76729090 | 0.9818 | 0.7241 | 1.331 | C | 0.9057 | T | 0.98477 | 0.905177847 |
| RS1996371 | LOC390612 | 76743861 | 1.177 | 0.9981 | 1.387 | G | 0.05274 | G | 0.000124 | 0.00017294 |
| RS12594550 |  | 76746092 | 0.9188 | 0.7157 | 1.179 | C | 0.5063 |  |  |  |
| RS8032156 |  | 76751553 | 0.9619 | 0.7692 | 1.203 | G | 0.7332 |  |  |  |
| RS1021071 |  | 76755234 | 1.195 | 1.014 | 1.41 | G | 0.03397 |  |  |  |
| RS4887077 |  | 76765419 | 1.177 | 0.9813 | 1.412 | T | 0.0789 |  |  |  |
| RS11072793 |  | 76793497 | 0.9063 | 0.7427 | 1.106 | A | 0.3325 |  |  |  |
| RS7182567 | LOC646938, LOC648189 | 76832109 | 0.9148 | 0.7464 | 1.121 | G | 0.3908 |  |  |  |
| RS8024939 |  | 76836292 | 0.9492 | 0.7682 | 1.173 | C | 0.6295 |  |  |  |
| RS1021069 | ADAMTS7 | 76860811 | 0.9317 | 0.6721 | 1.292 | G | 0.6711 |  |  |  |
| RS1994017 | ADAMTS7 | 76867361 | 0.8666 | 0.7195 | 1.044 | C | 0.1317 |  |  |  |
| RS2277547 | ADAMTS7 | 76869486 | 0.9224 | 0.7599 | 1.12 | T | 0.4139 |  |  |  |
| RS2277545 | ADAMTS7 | 76870646 | 1.207 | 1.011 | 1.44 | G | 0.03776 |  |  |  |
| RS3743057 | ADAMTS7 | 76876062 | 0.8367 | 0.6928 | 1.01 | G | 0.06403 |  |  |  |
| RS3825807 | ADAMTS7 | 76876166 | 1.203 | 1.019 | 1.421 | C | 0.02937 |  |  |  |
| RS7182809 | ADAMTS7 | 76892161 | 0.8294 | 0.6617 | 1.04 | C | 0.1047 |  |  |  |
