# Supplementary material for: A Genome-Wide Association Study in Chronic Obstructive Pulmonary Disease (COPD): Identification of Two Major Susceptibility Loci
Source: PLoS Genet. 2009 Mar 20;5(3):e1000421. doi: 10.1371/journal.pgen.1000421 (PMC2650282; doi:10.1371/journal.pgen.1000421)
Supplement: Table S3 — Results of the unadjusted analyses of the Bergen, ICGN and NETT/NAS populations. (0.04 MB DOC) [file pgen.1000421.s006.doc]

**Supplementary Table 3. Results of the unadjusted analyses of the Bergen, ICGN and NETT/NAS populations**

| Chr | SNP id | Bergen Case Control Population | | | ICGN Population | | NETT/NAS | |  |  |
| --- | --- | --- | --- | --- | --- | --- | --- | --- | --- | --- |
|  |  | Odds Ratio | Risk Allele | P value | p value (FBAT) | Risk Allele | P value | Risk Allele | Combined P value ^*^ | Gene/ Nearest Gene |
| 15 | rs8034191 | 1.312 | C | 0.0002 | 7.86 × 10 ^-7^ | C | 2.0 × 10 ^-5^ | C | **1.86** × **10 ^-12^** | NP_001013641.2 |
| 15 | rs1051730 | 1.300 | T | 0.0004 | 1.40 × 10 ^-6^ | T | 0.00025 | T | **6.56** × **10 ^-11^** | CHRNA3 |
| 5 | rs9686327 | 1.567 | A | 1.36x10 ^-6^ | 0.035 | A | 0.068 | G^*^ |  | ANKH |
| 5 | rs735243 | 1.590 | T | 3.10Ex10 ^-6^ | 0.044 | T | 0.0072 | C^*^ |  | ANKH |
| 2 | rs6720264 | 1.372 | A | 3.30x10 ^-5^ | 0.022 | A | 0.52 | A | 4.72 × 10 ^-5^ | ACVR1 |
| 4 | rs1828591 | 0.804 | A | 0.002 | 0.015 | A | 0.0014 | A | 6.81 × 10 ^-7^ | HHIP |
| 4 | rs13118928 | 0.804 | A | 0.002 | 0.017 | A | 0.0016 | A | 8.57 × 10 ^-7^ | HHIP |
| 10 | rs2488825^†^ | 1.364 | A | 0.0003 | 0.041 | A |  |  |  |  |

^*^ Fisher’s combined probability test was applied to combine the P-values from Bergen Cohort, ICGN cohort and NETT/NAS study.

P values in bold are above the genome-wide significance level (p < 1.01x10^-7^)
