# Supplementary material for: A Genome-Wide Association Study in Chronic Obstructive Pulmonary Disease (COPD): Identification of Two Major Susceptibility Loci
Source: PLoS Genet. 2009 Mar 20;5(3):e1000421. doi: 10.1371/journal.pgen.1000421 (PMC2650282; doi:10.1371/journal.pgen.1000421)
Supplement: Text S1 — Supplementary materials. (0.05 MB DOC) [file pgen.1000421.s007.doc]

**Supplementary Materials**

**Methods**

**S1 Quality control of data flow**

The following quality control steps were taken to make sure genotypes were correctly called.

1. Infinium BeadStudio Raw Data Analysis

All samples are brought into a single BeadStudio file, and using the standard Illumina cluster file an evaluation of the clustering of all samples is made. If a large number of samples do not fit the cluster for a random set of SNPs, a separate BeadStudio file is made for these samples. Once all files are made, any sample that has very low intensity or a very low call rate using the Illumina cluster (<95%) is deleted. All SNPs that have a call frequency below 100% are then reclustered. Any sample that is below a 98% call rate after the reclustering is deleted. Next, a “1% rule” is applied where all SNPs that have a call frequency below 99% are deleted. Any SNPs where more than 1% of samples are not called or are ambiguously called are deleted. We have shown (unpublished data) that SNPs with many samples not called (or potentially miscalled) can lead to false positives in statistical associations.

The reclustering step creates SNP calling errors, but we have identified a procedure to prevent the errant calls from being released in the final report. The SNP data is screened within BeadStudio by looking at two criteria. First, all SNPs with a cluster separation value below 0.3 are manually checked to ensure correct calls. Many of these SNPs can be manually fixed, but some have to be deleted. Next, any SNP (excluding X chromosome SNPs) with a Het Excess value between -1.0 to -0.1 and 0.1 to 1.0 are evaluated to determine if the raw and normalized data show a clean call. Any SNP cluster that doesn’t appear normal is deleted. This includes SNPs that appear to show a deletion (hemizygotes and homozygous deletion). The rationale is to avoid artifacts from either the chemistry or an interfering SNP during hybridization.

These procedures resulted in a success rate of genotyping calls ranging from 97.5%-99% in the different files (13,709-5355 deleted SNPs). Two percent of samples were selected randomly to be genotyped twice independently for quality purpose. The concordance rate for duplicate genotyping was 99.99%.

2. Minor allele frequency (MAF) check for data handling accuracy

This step performs a basic check of the data accuracy on the data flow pipeline from the output of the Illumina genotyping facility to the analytical process. We checked the MAF report from PLINK software [1] (http://pngu.mgh.harvard.edu/~purcell/plink/) against the original locus report generated by genotyping facility. We checked that the two MAF reports match exactly.

3. Specification of sex

This step performs a check on the sex specification obtained from the phenotype database, using the observed genotypes of SNPs on chromosome X and Y. All individuals who were marked as “male” but with significant amount of heterozygous X genotypes (>=1%), or who were marked as “female” but with high frequency of homozygous X genotypes (>=80%) or Y genotype readings, were individually inspected against original data source. If no satisfactory correction could be obtained these individuals were excluded from further analyses.

4. Cryptic relatedness

This step performs a check on the cryptic relatedness between study participants. We estimated the sharing of genetic information by estimating identity by descent (IBD) using the PLINK software. All pairs of DNA samples showing
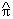
>=0.125 (estimated proportion of alleles IBD) were individually inspected and one sample in each pair was excluded from further analyses.

5. Genotype missing

This step performs a check whether the genotype missing is skewed towards high or low phenotype values and hence may give rise to spurious association *P*-values. We used PLINK software to perform this check on the top SNPs discussed in the paper. No genotype data violated this check.

6. Low MAF

We removed all SNPs with a MAF<0.002. This criterion ensured that at least 6 individuals of the rare genotype are present in the dataset, to control for error in the estimation of asymptotic *P*-values (as alleles with MAF this low or lower have no chance of approaching significance)

7. Hardy-Weinberg Equilibrium (HWE)

This step performs a check whether the observed genotype data deviate from HWE. We performed this check using PLINK software on the top SNPs. We defined a deviation from HWE with a criterion of *P*-value less than 0.05. Any SNPs that were of potential interest but failed this test were then subject to step 8: recheck of the genotyping quality and if necessary, were subject to a quality check using an independent platform, for example, using TaqMan assay.

8. Recheck of the genotyping quality

The top SNPs showing significant association were subject to a double check for their genotyping quality. This is an individual recheck on the raw and normalized data to be sure that it is called correctly as described in the “*Infinium BeadStudio Raw Data Analysis*” process. No genotype data violated this check.

**S2 Modified EIGENSTRAT method to control for stratification**

This method derives the principal components of the correlations among gene variants and corrects for those correlations in the association tests. In principle, the principal components in the analyses should reflect population ancestry. We have noticed however that some of the leading axes appear to depend on other sources of correlation, such as sets of variants near one another that show extended association. We have documented the potential for inversions to create this effect and it may be created by other causes of extended LD as well. For this reason we inspected the SNP ‘loadings’ for each of the leading axes to determine if they depended on many or relatively few SNPs, as would be expected if the given axis reflected population ancestry or a more localized LD effect respectively.

We selected EIGENSTRAT axes for use as covariates to adjust for ancestry in subsequent linear regression analyses as follows.

1. To find EIGENSTRAT axes, we started with autosomal SNPs with MAF>0.01.

2. On inspection of SNP loadings for each PC axis (the “gamma” coefficients of Price et al [2], we found several of the top axes to be dominated by a small number of SNPs all mapping to the same region of the genome. For example, one axis was found to be dominated by SNPs mapping to a region of chr8p22-23.1 coinciding with a known inversion polymorphism.

3. To correct for these LD effects, and ensure that EIGENSTRAT axes reflected only effects that applied equally across the whole genome (as ancestry effects should), we re-applied principal components analysis to a reduced SNP set in which (i) certain known high LD regions were excluded (chr8:8000000..12000000, chr6:25000000..33500000, chr11:45000000..57000000, chr5:44000000..51500000); (ii) SNPs were thinned using the “--indep-pairwise” option in PLINK, such that all SNPs within a window size of 1500 (step size of 150) were required to have r2<0.2; (iii) Each SNP was regressed on the previous 5 SNPs, and the residual entered into the PCA analysis, as suggested by Patterson et al [3].

4. Inspection of SNP loadings on all axes deemed significant by the Tracy-Widom method of Patterson et al [3], using Q-Q plots against Normal expectation, now revealed no axes dominated by single high-LD regions of the genome.

5. Tracy Widom tests nominated the first 12 resulting PC axes as significant (p<0.05). We therefore assessed significance using the 12 principal components emerging from the EIGENSTRAT analyses as covariates in a logistic regression model.

**S3 Regression analyses**

Additional covariates included in this model included sex, age, current smoking status, and pack-years. Logistic regression models [1] were used to test the genetic effect of each polymorphism from the markers that passed the quality control check. An additive model was assumed.

For all the SNPs that passed the quality control, we then performed a pair-wise linkage disequilibrium screen to prune out perfect-LD SNPs in our data set (r^2^=1), that is, for each pair of SNPs with r^2^=1, we kept only one of them in the analysis. For this screen we used the PLINK software with command: "--indep-pairwise 1500 100 1.0 --geno 0.1-mind 0.1 -maf 0.002". The 493,609 independent (pair-wise r^2^<1) SNPs generated from this procedure were then subject to the regression analysis.

**S4 Combined P value**

Fisher’s combined probability test was applied to combine the P-values from Bergen Cohort, ICGN cohort and NETT/NAS cohort. [(-2ln (P _Bergen_) + (-2ln(P _ICGN_) +(-2ln (P _NETT/NAS_) ~ *χ*^2^, _df=6_)].

Reference List

1. Purcell S, Neale B, Todd-Brown K, Thomas L, Ferreira MA, Bender D, Maller J, Sklar P, de Bakker PI, Daly MJ, Sham PC (2007) PLINK: a tool set for whole-genome association and population-based linkage analyses. Am J Hum Genet 81: 559-575.

2. Price AL, Patterson NJ, Plenge RM, Weinblatt ME, Shadick NA, Reich D (2006) Principal components analysis corrects for stratification in genome-wide association studies. Nat Genet 38: 904-909.

3. Patterson N, Price AL, Reich D (2006) Population structure and eigenanalysis. PLoS Genet 2: e190.
